# Impact of Contralateral Implant Placement in Unilateral Implant-Based Postmastectomy Breast Reconstruction: A Single Center Retrospective Cohort Study

**DOI:** 10.3390/jcm15010375

**Published:** 2026-01-04

**Authors:** Salvatore D’Arpa, Giuseppe Antonio D’Amico, Giulio Jad Jaber, Michele Rosario Colonna, Massimo David

**Affiliations:** 1 Plastic and Reconstructive Surgery, La Maddalena Cancer Center, 90146 Palermo, Italy; 2 Department of Plastic and Reconstructive Surgery, University Hospital of Messina AOU Gaetano Martino, 98125 Messina, Italy; 3 Unit of Plastic and Reconstructive Surgery, Department of Experimental and Clinical Medicine, Magna Graecia University, 88100 Catanzaro, Italy; 4 Division of Plastic and Reconstructive Surgery, Department of Surgery, China Medical University Hospital, Taichung 40447, Taiwan; 5 Department of Human Pathology and Diseases of the Adult, the Child and the Adolescent, University of Messina, 98125 Messina, Italy; 6 Oncologic Breast Surgery, La Maddalena Cancer Center, 90146 Palermo, Italy

**Keywords:** breast reconstruction, contralateral implant, implant-based reconstruction, breasts aesthetic unit

## Abstract

**Background/Objectives**: Investigating how placement of a contralateral breast implant in the context of unilateral implant-based breast reconstruction influences aesthetic and patient-reported outcomes. **Methods**: A retrospective analysis was performed on a single-center prospectively maintained database (January 2021–March 2025) including patients who underwent unilateral implant-based breast reconstruction in association with a contralateral implant placement or not. Exclusion criteria were bilateral implant-based reconstruction or autologous reconstruction, follow-up of less than 6 months and missing data. Demographics and complications were analyzed. Aesthetic outcomes were evaluated by independent blinded surgeons using the Kroll Scale, patient satisfaction was investigated with the BREAST-Q Reconstruction Module v2.0. Statistical analysis used the Student’s *t*-test, Multivariate regression analysis and Mann–Whitney U test with significance set at *p* < 0.05. **Results**: The study group included 21 patients (40.4%) who received a contralateral implant, while the control group included 31 patients (59.6%) who did not receive a contralateral implant. Patients who received contralateral implants showed a significant improvement in cosmetic outcomes evaluated with the Kroll Scale and a raw increment, without statistical significance, in BREAST-Q scores in all subsections investigated (Psychosocial well-being, Sexual well-being and Satisfaction with breasts). **Conclusions**: Placing a contralateral breast implant in the context of unilateral implant-based breast reconstruction significantly improves aesthetic outcomes and correlates with higher patient satisfaction scores for the reconstructed breast, compared to placing no contralateral implant, without increasing the overall complication rate.

## 1. Introduction

Unilateral implant-based breast reconstruction can lead to noticeable asymmetries due to differences in breast movement, consistency, and response to gravity, as well as distinct changes over time of the reconstructed breast compared with the contralateral breast.

Although a satisfactory unilateral breast reconstruction can be achieved with a unilateral implant, it is essential to consider both breasts as part of a unified thoracic aesthetic unit. The contralateral breast should therefore also be evaluated and, if necessary, adjusted to achieve overall symmetry and harmony of the chest. The breast is a component of the chest, which is made of two breasts, not just one. If we consider the chest as a single aesthetic unit, a unilateral implant-based breast reconstruction fails to comply with one of the basic principles of plastic surgery: to reconstruct “like with like” [1,2] since the breast is reconstructed with something very far from being like tissue.

Autogenous breast reconstruction allows to get very close to a like with like reconstruction, while unilateral implant-based reconstruction does not. Besides this, unilateral implant-based reconstruction seldom allows to achieve a symmetrical reconstruction, since creating a ptotic contour and satisfactory symmetry can be challenging and patient and surgeon expectations are rarely met [3].

After unilateral implant-based breast reconstruction, some patients are disappointed by the result: while some of these request a contralateral implant to balance the result by improving symmetry, others decline surgery and, despite disappointment, choose to live with it to avoid further surgeries.

We believe that the chest must be considered as a single aesthetic unit including two breasts: therefore we have started offering a contralateral implant to patients undergoing unilateral implant-based breast reconstruction, regardless of the type of mastectomy, reconstruction (fully heterologous or hybrid) and contralateral procedure (nothing, mastopexy or breast reduction). The rationale is to reconstruct two breasts, one chest, as similar as possible not only in shape, volume and form, but also in consistency and feeling. To achieve this, both breasts should be made—whenever possible—of a similar proportion of implant and autogenous tissue.

The aim of this study is to evaluate the impact of placing a contralateral implant in unilateral implant-based breast reconstruction by comparing outcomes of patients who underwent implant-based breast reconstruction with or without a contralateral breast implant. Aesthetic outcomes were blindly rated by independent observers based on Kroll Scale assessment. Patients’ satisfaction and their postoperative health-related quality of life (HRQoL) have been evaluated with the BREAST-Q questionnaire (reconstruction module and its subcategories).

## 2. Materials and Methods

A retrospective observational study was conducted using prospectively collected data from a single institutional database. Patients who underwent postmastectomy implant-based breast reconstruction between January 2021 and March 2025 were identified from the prospectively collected database.

Patients who received bilateral mastectomy, contralateral prophylactic mastectomy or unilateral autologous breast reconstruction with contralateral prosthetic augmentation were excluded. Patients with follow-up of less than 6 months from completion of breast reconstruction and symmetrization procedures were excluded from the study. Similarly, patients with missing data relevant to the data analysis were excluded from the study.

Fifty-two patients were included. The study group included 21 patients who underwent unilateral implant-based postmastectomy breast reconstruction with contralateral implant placement.

The control group, matched for clinical and operative variables, consisted of 31 patients who underwent unilateral implant-based postmastectomy breast reconstruction without contralateral implant placement.

Individual medical records were reviewed to compare demographic data, co-morbidities, type of mastectomy (Nipple sparing mastectomy, NSM; Skin sparing mastectomy, SSM; Skin reducing mastectomy, SRM), mastectomy specimen weight, implant size and revision operations, infection, skin flap necrosis, seroma/hematoma, capsular contracture, implant malposition and implant rupture.

Skin flap necrosis has been classified into “minor”, if managed conservatively, or “major” if managed surgically. Seroma and hematoma were defined as an accumulation of fluid or blood, addressed through aspiration or surgical treatment. Capsular contracture was only considered when staged as Baker class III or IV. Implant malposition was identified as an implant displacement requiring surgical correction. Implant rupture was included if confirmed by magnetic resonance imaging. Breast reconstruction failure was defined as the removal of a prosthetic device or flap without any further reconstruction.

Postoperative outcomes were evaluated by four independent surgeons (two male surgeons and two female surgeons who were not involved in the study and did not know the patients). The evaluators were blinded to the procedure and to the aim of the evaluation. The evaluators were only asked to score the results without any information on the purpose or the procedures performed. Each evaluator scored the results on his/her own to avoid any inter-evaluator influence. To obtain more objectivity, only one plastic surgeon was involved, the others were general surgeons (one of them being an oncologic breast surgeon). The observers were asked to review and grade results based on preoperative and postoperative photographs of patients without knowing what procedure had been performed. The reviewers were blinded to the type of procedure and the aim of surgery. Each patient was assigned a number. Scores were collected, anonymously connected to the patient’s code to relate the number to the group and analyzed by a statistician who was blinded to the type of procedure as well. The Kroll Scale was used as the evaluating tool [4]. Results were graded from 1.0 (excellent) to 4.0 (poor) according to the scale in the following subsections: Symmetry, Shape and Ptosis. Scars were not considered since they were considered independent of the type of reconstructive procedure performed but only related to the mastectomy incision and thus irrelevant to the object of the study. An overall evaluation score has been assigned to assess outcomes of the reconstruction as a whole.

Patients’ satisfaction was investigated with the BREAST-Q v2.0 reconstruction module that included the following subsections: Psychosocial well-being, Sexual well-being and Satisfaction with breasts. The BREAST-Q reconstruction module is a well-established and validated, patient-reported outcome tool that has the aim of analyzing health-related quality of life and satisfaction among patients who underwent breast reconstruction. All responses to the questionnaire are aggregated and the conversion to a standardized 0–100 scale is performed. Higher scores reflect greater satisfaction or enhanced quality of life [5,6].

Statistical analysis has been performed on the database. Differences between the study and the control group were assessed using the Student’s *t*-test and a Multivariate Linear Regression that used the postoperative BREAST-Q score as the dependent variable and included contralateral implant (yes/no), age, BMI, and radiation (yes/no) as independent predictors to control potential confounding factors. Kroll Scale ordinal data have been analyzed with the non-parametric Mann–Whitney U Test. The assumption of normality was verified using the Shapiro–Wilk test. Statistical analyses were performed using GraphPad Prism 10.5.0 version software for Windows, with the level of significance set at *p* < 0.05.

## 3. Results

A total of 52 patients who underwent unilateral implant-based breast reconstruction have been enrolled in this study and divided into two different groups based on the presence or not of a contralateral implant. The study group included 21 (40.4%) patients who received a contralateral implant as part of the symmetrization procedure. The control group included 31 patients (59.6%) who have not received a contralateral implant. Patients who received a contralateral implant were younger (50.65 y.o. vs. 53.18 y.o.) and had a lower BMI compared with the control population (24.52 kg/m^2^ vs. 25.87 kg/m^2^).

Nipple sparing mastectomy has been the most commonly performed type of mastectomy in both groups. The average mastectomy weight was higher in the unilateral implant group (357.58 g vs. 347.38 g) and similarly the implant volume in the reconstructed breast was higher (391.22 cc vs. 385.23 cc). The mean contralateral implant volume was 225.23 cc ± 70.66 cc.

Adjuvant radiation therapy was performed by 25.8% of the patients in the contralateral implant group and by 38.09% of the patients in the unilateral implant group. Other demographic and clinical variables such as diabetes, hypertension and chemotherapy, either in neoadjuvant or adjuvant settings, have been considered and recorded (see Table 1).

The overall complication rate was 38.46% (see Table 2). Skin flap necrosis, either minor or major, occurred in 17.31% and resulted in the most common complication followed by infection, whose rate was about 11.54% overall. Other complications such as seroma, implant malposition and capsular contracture have been recorded. Only two cases of reconstructive failure were observed among the entire cohort.

Minimum follow-up time to BREAST-Q assessment was 6 months after the end of reconstruction. The average follow-up was 1.58 years for the contralateral implant group, while the average follow-up of the unilateral implant group was 1.85 years. The average follow-up among the entire cohort was 1.72 years.

Patients in the contralateral implant group received better scores in all subsections of the Kroll Scale. A statistically significant mean increase was observed in the symmetry, shape, and ptosis scores across the individual subsections of the contralateral implant group when compared with the unilateral implant group. Regarding the overall aesthetic evaluation scores, the study group scored higher than the control group, although the difference was not statistically significant. The results of the Kroll Scale and its items are reported in Table 3.

Patients who received contralateral implant placement registered higher BREAST-Q scores in the postoperative satisfaction with the breast (65.315 vs. 60.218) subsection even though the difference between the two groups was not statistically significant (*p* = 0.247). Psychosocial well-being and Sexual well-being sections registered slightly higher scores in the study group and were not statistically significant (see Table 4). The Multivariate Linear Regression analysis showed that, while the presence of a contralateral implant was not an independent predictor of BREAST-Q score (*p* = 0.520), BMI and radiation therapy were significantly associated with lower satisfaction scores (*p* = 0.0078 and *p* = 0.0115, respectively); results are reported in Table 5.

## 4. Discussion

The results of this study demonstrate that, among patients who undergo unilateral implant-based postmastectomy breast reconstruction, those who have a contralateral implant have better aesthetic outcomes and are more satisfied with their breasts than those who have no contralateral implant.

In order to objectively evaluate results, we asked four independent observers to evaluate results. Picture evaluation by four noninvolved and blinded observers showed better scores overall and in all single areas of evaluation for patients who received a contralateral implant compared to those who did not, showing that placement of a contralateral implant improves aesthetic outcomes.

Patients who received a contralateral implant showed a raw increase of 5 points in BREAST-Q scores regarding postoperative satisfaction with their breasts, compared to patients who had no contralateral implant. Although the difference was not statistically significant (*p* 0.247), it is clear that patients who undergo unilateral implant-based breast reconstruction tend to be more satisfied with their breasts when a contralateral implant is placed, compared to those who have an implant only in the reconstructed breast.

The other two BREAST-Q subsections, Psychosocial well-being and Sexual well-being, deserve a separate analysis: in these sections we recorded interesting results (Table 3). The scores showed only a small improvement in the study group with a raw increment of 1 or less, not statistically significant. Thus, we conclude that these areas are not significantly influenced by placement of a contralateral implant. Worthy of note is the fact that, when compared to the values reported by Razdan et al., the scores obtained by our population appear comparable in value, but the areas are inverted. In contrast to our study, Razdan et al. registered higher scores in the Psychosocial well-being area than in the Sexual well-being area. We infer that such differences might be related to geographic and cultural differences rather than being treatment-related [7].

Based on the results of our study, adding a contralateral implant does not increase complication rates, ruling out any potential risk increase and any influence of complications on the differences registered in BREAST-Q scores. The two groups in our study showed comparable complication rates (38.10% for the contralateral implant group and 38.71% for the unilateral implant group). Reconstructive failures were relatively rare (3.85%) and, again, comparable between groups (one case recorded for each group). After breaking down complication rates, we have found out that 17.31% (nine patients) are linked to mastectomy flap necrosis. Reconstruction-related complications are in line with the current literature [8,9].

Factors such as Age, Marital status, Education and Income remain independently associated with BREAST-Q scores [10,11]. Patients who received contralateral implants were younger and had a lower BMI compared to those who did not. Lower BMIs confirm what was already reported by Liu et al., that slender patients are candidates for contralateral implants as a symmetrization procedure [12]. Slender patients or patients with small breasts wgi receive a contralateral implant have higher satisfaction scores compared to patients who do not receive a contralateral implant [13,14,15]. Moreover, both BMI and Radiation therapy were significantly associated with lower BREAST-Q postoperative satisfaction scores (*p* = 0.0078 and *p* = 0.0115, respectively). In summary, our data are in line with the current literature showing that radiation therapy is consistently associated with lower BREAST-Q scores [16].

**Figure 1:**
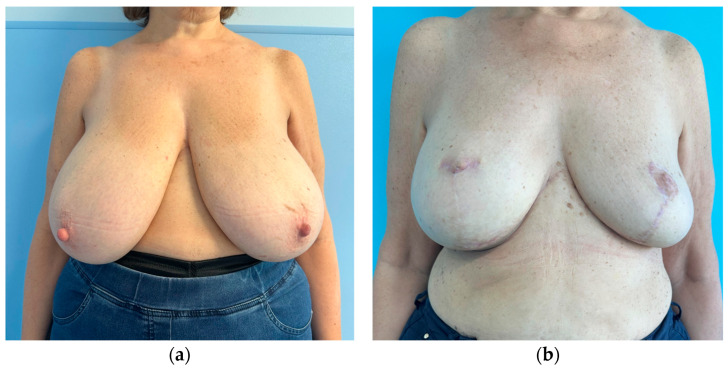
Preoperative (**a**) and one-year postoperative (**b**) result of a patient from the control group (no contralateral implant) who underwent right skin reducing mastectomy (1500 g of mammary gland removed), pre-pectoral implant breast reconstruction and contralateral breast reduction (1000 g of mammary gland removed). The right implant is a polyurethane-coated implant, short base, high projection, 625 cc.

**Figure 2:**
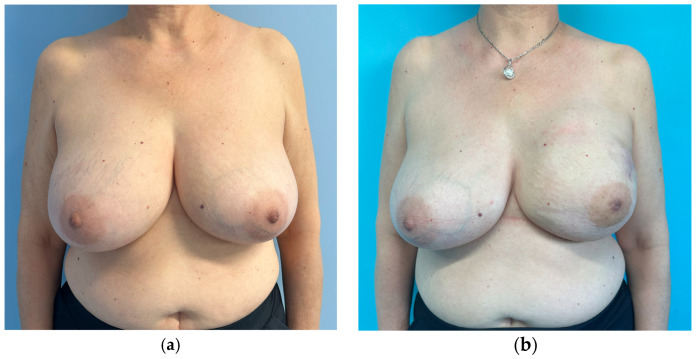
Preoperative (**a**) and six months postoperative (**b**) result of a patient from the control group (no contralateral implant), who underwent left nipple-sparing mastectomy and pre-pectoral direct-to-implant breast reconstruction with a polyurethane-coated, round base, high projection 495 cc implant (mastectomy weight was 500 g).

**Figure 3:**
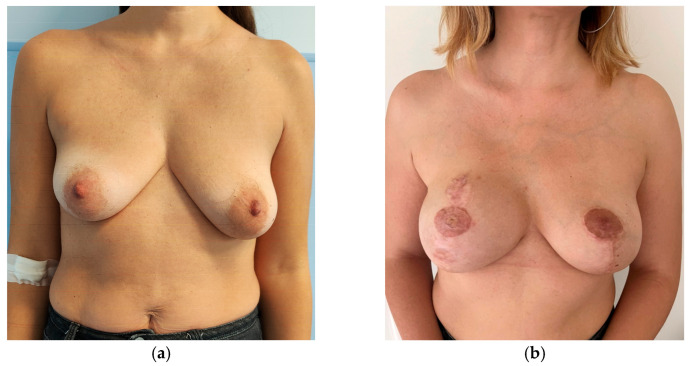
Preoperative (**a**) and two-years postoperative (**b**) result of a patient from the study group (contralateral implant) after right mastectomy followed by pre-pectoral implant-based breast reconstruction and contralateral breast reduction associated with placement of a pre-pectoral implant. The right implant is a polyurethane-coated implant, short base, extra high projection, 500 cc. The left implant is a polyurethane-coated implant, short base, low projection, 240 cc.

**Figure 4:**
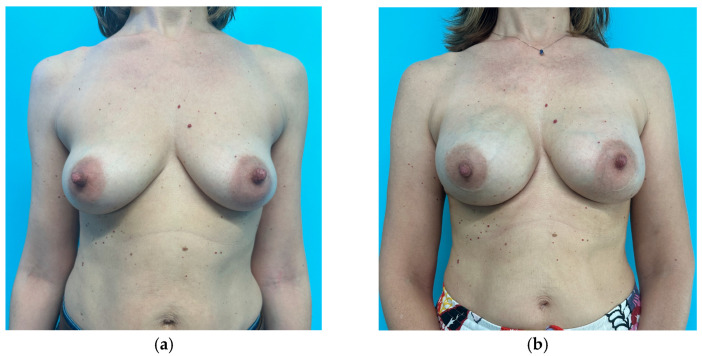
Preoperative (**a**) and one-year postoperative (**b**) result of a patient from the study group (contralateral implant) who underwent right nipple-sparing mastectomy through a sub-mammary incision and pre-pectoral implant-based breast reconstruction with contralateral breast augmentation. The right implant is a polyurethane-coated implant, round base, high projection, 425 cc. The left implant is a polyurethane-covered implant, round base, low projection, 165 cc.

Analysis of Kroll scores assigned by the independent panel of surgeons highlights that scores received by patients who had no symmetrization procedure, whether slender or not, are very low. To an objective observer what was considered a good result by the operating surgeon, might receive very low scores (Figure 1, Figure 2, Figure 3 and Figure 4). The former aspect supports our choice of an independent panel of observers to evaluate the aesthetic outcomes.

Before the results of this study, we used the following criteria:

1. There was an absolute indication to placement of a contralateral implant when no other symmetrization procedure was planned (mastopexy or breast reduction) irrespective of the size of the breast.
2. Patients who undergo contralateral mastopexy alone benefit from the association of a contralateral implant and thus this results in an absolute indication as well. The option of placing a contralateral implant was discussed with the patient and evaluated case by case.
3. In patients who need a breast reduction, the indication for implant placement can be considered relative and should be evaluated case by case.

Partially supported by the existing literature [7,12,17,18], and based on the above-mentioned findings, we have revised our decisional algorithm.

Placement of a contralateral implant seems to warrant better stability of results over time [18]. Since placing an implant complies better with the principle of reconstructing like-with-like by reconstructing two breasts that are both hybrids, made of autogenous tissue and implant, we advocate use of contralateral implants in all cases as well. A 2002 study by Losken et al. reported that an implant placement represents the most common symmetrization procedure in the setting of a postmastectomy implant-based reconstruction while breast reduction is more often associated with autologous breast reconstruction [19].

Nowadays we consider any patient undergoing unilateral implant-based postmastectomy breast reconstruction as a candidate for contralateral implant placement, regardless of the type of symmetrization procedure required (none, mastopexy, or breast reduction).

This introduces indication to placement of a contralateral implant in all cases of unilateral prosthetic postmastectomy breast reconstruction. We would like to introduce the concept of a thoracic aesthetic subunit in breast reconstruction, since calling it “breast reconstruction” does not necessarily involve chest restoration and rehabilitation of women affected by breast cancer and mutilated by the mastectomy necessary to cure cancer. Chest reconstruction shall be considered to restore integrity, wholeness and well-being of the woman affected. The focus shall be moved from the breast to the woman. In this light, chest reconstruction must involve bilateral implant placement to balance the two breasts to make them as like each other as possible. In the context of implant-based breast reconstruction, pursuing this goal makes contralateral implant placement a necessary step in achieving balanced reconstruction of a woman’s chest and wholeness.

This study has some flaws: the number of patients recruited is relatively low and this might justify failure to reach statistical significance, which is the second flaw. The blindness in evaluation and judgement was maintained by all the staff involved throughout the study.

Follow-up is not long enough to conclude about behavior over time. However, a previous large cohort study made by Barone et al. demonstrated, thanks to a validated patient-reported outcome measure scale (BREAST-Q Reconstruction module), the stability of the contralateral-based implant procedure as the most effective for achieving lasting results, yielding statistically significant scores both in the Psychosocial well-being and in the Physical well-being subsections [18].

This patient obtained the following scores at the Kroll Scale: 2.25 for Symmetry, 2 for Ptosis, 2.5 for Shape and an Overall evaluation of 2.25.

This patient obtained the following scores at the Kroll Scale: 3 for Symmetry, 2.5 for Ptosis, 3.25 for Shape and an Overall evaluation of 3.

This patient obtained the following scores at the Kroll Scale: 1.25 for Symmetry, 1.75 for Ptosis, 1.5 for Shape and an Overall evaluation of 1.5.

This patient obtained the following scores at the Kroll Scale: 1 for Symmetry, 1.25 for Ptosis, 1.5 for Shape and an Overall evaluation of 1.25.

## 5. Conclusions

This study demonstrates that placement of a contralateral breast implant significantly improves aesthetic outcomes (Symmetry, Shape, and Ptosis) and correlates with higher patient satisfaction scores for the reconstructed breast, all without increasing the overall complication rate.

Since the introduction of the first silicone breast implant by Cronin and Gerow in 1962 [20], implant-based breast reconstruction has undergone remarkable evolution. The goal is no longer merely to reconstruct, but to reconstruct aesthetically. This concept applies to all forms of breast reconstruction, including autologous techniques [21]. When evaluating the breast, both breasts must always be considered; there is no such thing as breast reconstruction without taking the contralateral breast into account. From the preoperative to the postoperative phase, both breasts should consistently be evaluated. A preoperative photograph that does not include the contralateral breast is considered incomplete; an operative field that excludes one of the two breasts results as inappropriate; and a postoperative photograph without it is inadequate. In this light, the term chest reconstruction appears more appropriate than simply breast reconstruction.

Just as surgeons evaluate both breasts, so does the patient: she will always perceive two breasts. In the same way that Burget and Menick defined the aesthetic subunits of the nose, we believe that the chest should be regarded as a single aesthetic unit [1].

Therefore, we advocate routine contralateral breast implant placement in all unilateral implant-based breast reconstructions as a recommendation that applies to fully prosthetic (direct-to-implant or two-stage) and hybrid (implant with autogenous tissue/lipofilling) techniques. This approach consistently yields superior aesthetic results and meets patient expectations more reliably by achieving chest reconstruction rather than breast reconstruction.

## Figures and Tables

**Table 1:** **Demographics andlinical characteristics.** NSM, *Nipple Sparing Mastectomy*; SSM, *Skin Sparing Mastectomy*; SRM, *Skin Reducing Mastectomy*.

	Subgroup	Unilateral Breast Implant (Mean ± SD)	Bilateral Breast Implant (Mean ± SD)
Patient no.		31	21
Age, years		53.18 ± 9.21	50.65 ± 7.52
BMI, kg/m^2^		25.87 ± 5.15	24.52 ± 7.31
Diabetes			
	Yes	6	3
	No	25	18
Smoke			
	Yes	11	9
	No	20	12
Hypertension			
	Yes	6	5
	No	25	16
Mastectomy type			
	NSM	13	10
	SSM	9	7
	SRM	9	4
Mastectomy weight, g		357.58 ± 118.01	347.38 ± 95.48
Neoadjuvant chemotherapy			
	Yes	5	6
	No	26	15
Previous radiation therapy			
	Yes	0	1
	No	31	20
Adjuvant chemotherapy			
	Yes	15	11
	No	16	10
Adjuvant radiation therapy			
	Yes	8	8
	No	23	13
Implant size (reconstructed breast), cc		391.22 ± 126.02	385.23 ± 91.69
Contralateral implant size, cc		/	225.23 ± 70.66

**Table 2:** **Postoperative complications**.

	Subgroup	N (%)	Unilateral Breast Implant	Bilateral Breast Implant
			n (%)	n (%)
Patient no.		52 (100.00%)	31 (100.00%)	21 (100.00%)
Complications no.		20 (38.46%)	12 (38.71%)	8 (38.10%)
Hematoma/Hemorragia				
	Yes	1 (1.92%)	0 (0.00%)	1 (4.76%)
	No	50 (96.15%)	31 (100.00%)	20 (95.24%)
Skin flap necrosis				
	Yes—minor	6 (11.54%)	5 (16.13%)	1 (4.76%)
	Yes—major	3 (5.77%)	2 (6.45%)	1 (4.76%)
	No	43 (82.69%)	24 (77.42%)	19 (90.48%)
Seroma				
	Yes	1 (1.92%)	1 (3.23%)	0 (0.00%)
	No	50 (96.15%)	30 (96.77%)	21 (100.00%)
Infection				
	Yes	6 (11.54%)	2 (6.45%)	4 (19.05%)
	No	46 (88.46%)	29 (93.55%)	17 (80.95%)
Implant malposition				
	Yes	1 (1.92%)	0 (0.00%)	1 (4.76%)
	No	51 (98.08%)	31 (100.00%)	20 (95.24%)
Capsular contracture				
	Yes	1 (1.92%)	0 (0.00%)	1 (4.76%)
	No	51 (98.08%)	31 (100.00%)	20 (95.24%)
Implant rupture				
	Yes	0 (0.00%)	0 (0.00%)	0 (0.00%)
	No	51 (100.00%)	31 (100.00%)	21 (100.00%)
Reconstruction failure				
	Yes	2 (3.85%)	1 (3.23%)	1 (4.76%)
	No	50 (96.15%)	30 (96.77%)	20 (95.24%)

**Table 3:** **Kroll Scale—Outcome scores and statistical analysis (*p* < 0.05)**.

	Unilateral Breast Implant	Bilateral Breast Implant	*p*
	Mean (±SD)	Mean (±SD)	
Symmetry	2.297 (±0.853)	1.825 (±0.444)	0.037
Shape	2.375 (±0.886)	1.85 (±0.572)	0.022
Ptosis	2.187 (±0.903)	1.675 (±0.403)	0.017
Overall evaluation	2.312 (±0.850)	1.9 (±0.672)	0.069

**Table 4:** **BREAST-Questionnaire Reconstruction Module v.2.0.—Patient scores and statistical analysis (*p* < 0.05)**.

BREAST-Questionnaire Reconstruction Module Version 2.0	Unilateral Breast Implant	Bilateral Breast Implant	*p*
	Mean (±SD)	Mean (±SD)	
Satisfaction with Breasts (Postoperative)	60.218 (16.653)	65.315 (13.275)	0.247
Psyschosocial Well-Being (Preoperative)	37.947 (6.628)	38.093 (6.683)	0.938
Psychosocial Well-Being (Postoperative)	65.052 (16.899)	65.612 (14.634)	0.902
Sexual Well-Being (Preoperative)	30.210 (17.415)	31 (17.121)	0.872
Sexual Well-Being (Postoperative)	74.052 (25.968)	75.225 (25.468)	0.873

**Table 5:** **Multivariate Linear Regression analysis**.

Variable	β (Coefficient)	Robust SE	95% Confidence Interval	*p*-Value
Intercept	114.16	22.93	69.22–159.11	<0.001
Contralateral Breast Implant (yes)	(+3.24)	5.05	(−6.65)–(+13.14)	0.520
Age (years)	(−0.21)	0.33	(−0.87)–(+0.44)	0.522
BMI (kg/m^2^)	(−1.53)	0.58	(−2.66)–(−0.40)	0.0078
Radiation (yes)	(−19.77)	7.83	(−35.11)–(−4.43)	0.0115

## Data Availability

The original contributions presented in this study are included in the article. Further inquiries can be directed to the corresponding author.
